# Frequency of night shift and menstrual cycle characteristics in Japanese nurses working under two or three rotating shifts

**DOI:** 10.1002/1348-9585.12180

**Published:** 2020-11-19

**Authors:** Michinori Mayama, Takeshi Umazume, Hidemichi Watari, Sho Nishiguchi, Takuhiro Moromizato, Takashi Watari

**Affiliations:** ^1^ Department of Obstetrics and Gynecology Hokkaido University Graduate School of Medicine Sapporo Japan; ^2^ Department of General Internal Medicine Shonan Kamakura General Hospital Kamakura Japan; ^3^ Renal & Rheumatology Division Internal Medicine Department Okinawa Prefectural Nanbu Medical Center and Children's Medical Center Haebaru‐cho Japan; ^4^ Post Clinical Training Center Shimane University Hospital Izumo Japan

**Keywords:** irregular menstrual cycle, night shift, nurse, rotating shift working, secondary amenorrhea

## Abstract

**Objectives:**

In Japan, the prevalence of irregular menstrual cycles and its association with the frequency of night shifts have scarcely assessed. The present study aimed to evaluate the relationship between irregular menstrual cycles and the frequency of night shifts in Japanese female nurses.

**Methods:**

We conducted a cross‐sectional web‐based self‐administered questionnaire survey in 2019. An irregular menstrual cycle was defined as a cycle length of ≤21 days or ≥39 days at least a few times over the past year or amenorrhea for at least 3 months. We used Poison regression analysis with a robust error variance to calculate the prevalence ratios adjusted for age, body mass index, hospital size, and the department in which they worked.

**Results:**

A total of 1249 women were included, and 679 (54.4%) and 195 (15.6%) of them worked under two and three rotating shifts. The prevalence of irregular menstrual cycles was 24.8%, 37.4%, and 35.9% in the no night, two rotating, and three rotating shifts groups, respectively. While the frequency of night shifts had a dose‐responsive relationship with irregular menstrual cycles in the two rotating shifts group, it was not observed in the three rotating shifts group. However, the risk of work getting affected by dysmenorrhea or premenstrual symptoms increased in the three rotating shifts group.

**Conclusions:**

Over 30% of Japanese female nurses working under night shifts had irregular menstrual cycles. The high frequency of night shifts increased the risk of irregular menstrual cycles and secondary amenorrhea in the two rotating shifts group.

## 1 INTRODUCTION

The menstrual cycles is controlled by the cyclical secretion of reproductive hormones, including luteinizing hormone, follicle‐stimulating hormone, estrogen, and progesterone, which are regulated by the hypothalamus – pituitary – ovarian axis.^1^ The absence of menses ≥3 months is diagnosed as secondary amenorrhea. Over 50% of secondary amenorrhea cases were due to a disturbance of hypothalamic‐pituitary‐adrenal axis, which can lead to infertility.^2^ In addition, estrogen deficiency caused by secondary amenorrhea increases the risk of cardiovascular disease, osteopenia, and depression.^3^, ^4^, ^5^

Menstrual cycles are assessed by their length and regularity and are considered as a marker of reproductive health.^6^, ^7^ Disruption of the circadian rhythm during shift work affects the regulation of the hypothalamus‐pituitary‐ovarian axis and alters the menstrual cycles.^8^, ^9^ Studies using data from the Nurse's Health Study II (71 077 female nurses) and Nurse's Health Study III (6309 female nurses) reported that the incidence of irregular menstrual cycles were 11%‐19%, and that the age‐adjusted prevalence risk (PR) was 1.20‐1.34 for irregular menstrual cycles in women working rotating shift.^8^, ^9^

The Nurse's Health Study II showed that women of Asian ethnicity had a higher risk of irregular menstrual cycles than that of white people (PR: 1.38, 95% confidence interval [CI]: 1.19‐1.61).^8^ Polycystic ovary syndrome (PCOS) is a common endocrine disorder in women of reproductive age and irregular menstruation, and the polycystic ovary subgroup is relatively common in Asian ethnicities.^10^ In addition, genetic studies on PCOS reported that there is polymorphism in follicular stimulating hormone receptor gene, which plays key role in a menstrual cycle.^11^, ^12^ These might be the reasons for the higher risk of irregular menstrual cycles in Asian ethnicities.

The prevalence of dysmenorrhea and premenstrual symptoms exceed 70%, and dysmenorrhea can deteriorate women's quality of life.^13^, ^14^ However, the effect of rotating shifts on dysmenorrhea is still controversial. While the study on 113 women working rotating shift and 75 women working day shift reported that rotating shift was not associated with dysmenorrhea,^15^ a study on 420 Taiwanese nurses showed that a higher percentage of women with dysmenorrhea worked under three shift rotations than women without dysmenorrhea (91.3% vs 82.9%, *P* = .014).^14^

In each country, working hours and conditions vary. While night shift working hours are regulated within 12 hours in many counties, >90% of hospitals in Japan adopt a 16‐hour night shift as two rotating shifts.^16^ While a 16‐hour night shift may increase the risk of irregular menstrual cycle compared with a 12‐hour night shift; however, data are scarce regarding the incidence of irregular menstrual cycle among Japanese nurses. Differences in ethnicities and working conditions could affect the development of irregular menstrual cycles. Therefore, the present study aimed to determine the prevalence of irregular menstrual cycles and dysmenorrhea in female nurses working under rotating shifts in Japan and to evaluate the effect of night shifts on irregular menstrual cycles and dysmenorrhea.

## 2 METHODS

### 2.1 Study design and participants

**FIGURE 1:**
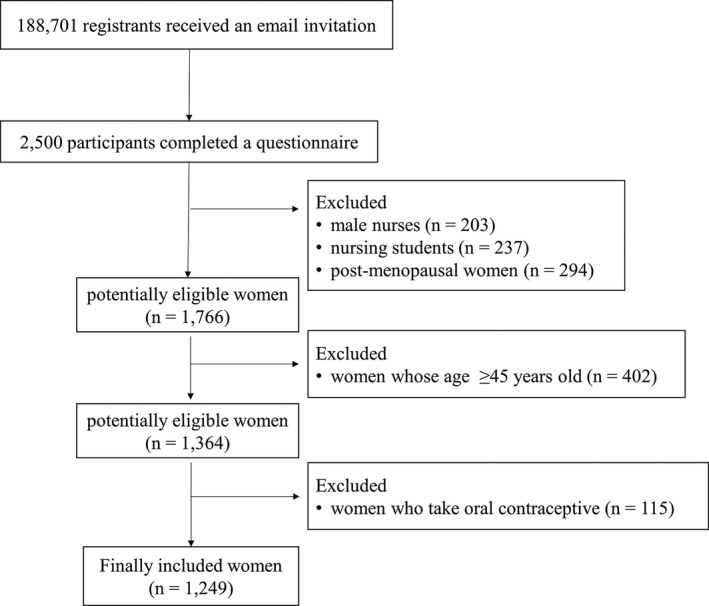
Schematic illustration of the patient enrollment procedure

We conducted a cross‐sectional study on nursing education and shift working in Japan using a web‐based self‐administered questionnaire. This survey was conducted from March to August 2019 and utilized the data related to shift working and menstrual irregularity in the survey. The study population was obtained from the registrants of “*Nurse senka plus*”, which is a website (https://nursepress.jp/) that provides information about nursing skills and medical topics for Japanese nurses. The questionnaire was published on the homepage of “*Nurse senka plus*” and an email invitation was sent to each registrant. Among the participants, 33 nurses won rewards by draw: 1 nurse won \10 000 ($95), 2 nurses won \5000 ($48), 10 nurses won \1000 ($9.5), and 20 nurses won \500 ($4.8). From a total of 188 701 registrants, 2500 nurses completed the questionnaire. We excluded male nurses, nursing students, and post‐menopausal women from the study. It is reported that the median age of menopause occurrence in Japanese women is 50.54 years.^17^ We excluded women age ≥45 years because irregular menstrual cycles sharply increase beginning from 5 years before menopause.^18^ In addition, women who took oral contraceptives were also excluded from the analysis; however, we assessed the reasons for oral contraceptive use among these women. A total of 1249 women were finally included in the analysis (Figure 1).

The institutional review board of Shimane University approved this study (20181018‐2), and completion of the web‐based questionnaires implied that informed consent was provided.

### 2.2 Data collection

The questionnaire collected the following data: weight, height, age (<30, 30‐39, 40‐44, 45‐49, ≥50 years old), bed number (no beds: clinic or nursing home, 1‐99 beds, 100‐299 beds, ≥300 beds), department in which they worked (out‐patient clinic, wards, intensive care unit or operating room, and emergency room), oral contraceptive use, reasons for oral contraceptive use (contraception, dysmenorrhea, irregular menstrual cycles, treatment for endometriosis, and others), night shift frequency per month over the past year (0, 1‐3 nights, 4‐5 nights, 6‐7 nights, ≥8 nights), and shift working patterns (two rotating shift or three rotating shift). Body mass index (BMI) was calculated using the weight and height and was categorized into four groups (<18.5, ≥18.5 to <25, ≥25 to <30, >30). In addition, participants provided information about their menstrual cycles over the past year (no irregular cycles, cycle length ≤21 days or ≥39 days at least a few times over the past year, absence of menses ≥3 months) and menstrual period (1‐2, 3‐7, ≥8 days). These categories were determined based on the definition of Japan Society of Obstetrics and Gynecology.^19^ We also asked the participants whether dysmenorrhea or premenstrual symptoms affected their work (no, sometimes, often).

### 2.3 Statistics

We used Stata/SE version 15.1 (StataCorp) to conduct all the analysis. Statistical significance among the three groups was calculated using chi‐square test. We used a Poisson regression analysis with a robust error variance to calculate the PR of irregular menstrual cycle, abnormal menstrual period, and the effects of menstrual related symptoms on work in relation to the frequency of night shifts, and women who did not work night shifts were included as a reference group. The number of women having 1‐3 night shifts per month in three rotating shifts and having ≥8 night shifts per month in two rotating shifts was small; therefore, we used different categories for the frequency of night shifts: 1‐3 nights, 4‐5 nights, and ≥6 nights per month for women working under two rotating shifts and ≤5 nights, 6‐7 nights, and ≥8 nights per month for women working under three rotating shifts. We created two models: model 1 was adjusted for age. and in model 2, BMI, hospital size, and the department in which women worked was added to model 1. We also created separate models for severe types of irregular menstrual cycles as well as for the effects of menstrual‐related symptoms on work.

## 3 RESULTS

Among the eligible participants, 874 women (70.0%) had night shifts: 679 women (54.4%) worked under two rotating shift and 195 women (15.6%) under three rotating shifts. Table 1 presents the characteristics and menstrual‐related variables based on the rotating shift patterns. While the prevalence of irregular menstrual cycles was 20.8% in women without night shift, 37.4% and 35.9% of the women working under two and three rotating shifts, respectively, suffered from irregular menstrual cycles. The rate of people who answered that dysmenorrhea or premenstrual symptoms affected their work was 62.9%, 68.2%, and 67.7% in women without night shifts, women working under two and three rotating shifts, respectively. and Table S1 and S2 show the characteristics and menstrual‐related variables stratified by frequency of night shifts per month over the past year in women with two or three rotating shifts. Although there was no significant difference, the prevalence of irregular menstrual cycles (cycle length ≤21 days or ≥39 days at least a few times: 29.3%, 30.6%, 34.2% in women working 1‐3, 4‐5, ≥6 night shifts per month, respectively; amenorrhea ≥3 months: 3.3%, 5.6%, 9.8% in women working 1‐3, 4‐5, ≥6 night shifts per month, respectively, *P* = .107) tended to be higher as the frequency of night shifts increased in women with two rotating shifts. On the other hand, the trend was not detected in women with three rotating shifts (cycle length ≤21 days or ≥39 days at least a few times: 35.7%, 30.0%, 27.3% in women working ≤5, 6‐7, ≥8 night shifts per month, respectively; amenorrhea ≥3 months: 10.7%, 7.5%, 2.0% in women working ≤5, 6‐7, ≥8 night shifts per month, respectively, *P* = .107).

**TABLE 1:** Characteristics and menstrual‐related variables based on the rotating shift patterns

	No night shifts (n = 375)	Two rotating shifts (n = 679)	Three rotating shifts (n = 195)	*P*‐value
Age (years old)				
<30	69 (18.4)	274 (40.4)	85 (43.6)	<.001
≥30 and <40	150 (40.0)	226 (33.3)	60 (30.8)	
≥40 and <45	156 (41.6)	179 (26.4)	50 (25.6)	
BMI (kg/m^2^)				
≥18.5 and <25	293 (78.1)	484 (71.3)	150 (76.9)	.090
<18.5	47 (12.5)	97 (14.3)	26 (13.3)	
≥25 and <30	23 (6.1)	73 (10.8)	11 (5.6)	
>30	12 (3.2)	25 (3.7)	8 (4.1)	
Hospital size				
>300 beds	107 (28.5)	381 (56.1)	119 (61.0)	<.001
100‐299 beds	94 (25.1)	226 (33.3)	64 (32.8)	
<100 beds	25 (6.7)	40 (5.9)	11 (5.6)	
Clinic, nursing home	149 (39.7)	32 (4.7)	1 (0.5)	
Department in which the women worked in				
Out‐patient clinic	240 (64.0)	63 (9.3)	8 (4.1)	<.001
Wards	118 (31.5)	530 (78.1)	157 (80.5)	
ICU, OR, ER	17 (4.5)	86 (12.7)	30 (15.4)	
Irregular menstrual cycles^a^	93 (24.8)	254 (37.4)	70 (35.9)	.001
Cycle length ≤21 d or ≥39 d at least a few times	78 (20.8)	212 (31.2)	59 (30.3)	
Amenorrhea ≥3 mo	15 (4.0)	42 (6.2)	11 (5.6)	
Abnormal menstrual period^b^	30 (8.0)	45 (6.6)	18 (9.2)	0.629
1‐2 d	9 (2.4)	15 (2.2)	4 (2.1)	
≥8 d	21 (5.6)	30 (4.4)	14 (7.2)	
Dysmenorrhea or premenstrual symptoms affected their work^c^	236 (62.9)	463 (68.2)	132 (67.7)	.429
Sometimes	199 (53.1)	379 (55.8)	108 (55.4)	
Often	37 (9.9)	84 (12.4)	24 (12.3)	

Note

Data are shown in n (%). Statistical significance was calculated using chi‐square test.

Abbreviations: BMI, body mass index; ER, emergency room; ICU, intensive care unit; OR, operating room.

^a^ Normal menstrual cycle length is >22 and <39 d.

^b^ Normal menstrual period is 3‐7 d.

^c^ Dysmenorrhea and premenstrual symptoms do not affect their work in a normal person.

Table 2 shows the association of rotating shift patterns and menstrual‐related variables. The results were consistent between model 1 and model 2. In model 2, the PR of irregular menstrual cycles was significantly higher in two rotating shifts (PR, 1.48; 95% CI, 1.16‐1.89) and three rotating shifts (PR, 1.43; 95% CI, 1.06‐1.92). Tables 3 and 4 present the relationship between the frequency of night shifts per month and menstrual‐related variables. In model 2, women working 4‐5 and ≥6 night shifts per month under two rotating shifts showed an increased risk of irregular menstrual cycles (PR, 1.41; 95% CI, 1.08‐1.83, and PR, 1.78; 95% CI, 1.34‐2.35). In addition, the risk of absence of menses ≥3 months significantly increased in women working ≥6 night shifts per month (PR, 2.39; 95% CI, 1.27‐4.50). In contrast, the risk of irregular menstrual cycles increased only in women working ≤5 night shifts per month (PR, 1.93; 95% CI, 1.29‐2.89) among women working under three rotating shifts. Although abnormal menstrual period and the effects of dysmenorrhea or premenstrual symptoms were not associated with the frequency of night shifts per month in women working under two rotating shifts, the risk of work getting affected increased in women working ≥8 night shifts per month under three rotating shifts (PR, 1.23; 95% CI, 1.02‐1.49).

**TABLE 2:** Association of the rotating shift patterns and menstrual‐related variables

	Shift patterns	Model 1		Model 2	
		PR	95% CI	PR	95% CI
Irregular menstrual cycles	No night shifts	Reference		Reference	
	Two rotating shifts	1.46	1.19‐1.79	1.48	1.16‐1.89
	Three rotating shifts	1.40	1.08‐1.82	1.43	1.06‐1.92
Amenorrhea ≥3 mo	No night shifts	Reference		Reference	
	Two rotating shifts	1.57	0.86‐2.84	1.32	0.74‐2.35
	Three rotating shifts	1.38	0.64‐3.00	1.29	0.61‐2.72
Abnormal menstrual period	No night shifts	Reference		Reference	
	Two rotating shifts	0.91	0.58‐1.44	0.73	0.44‐1.19
	Three rotating shifts	1.29	0.73‐2.28	1.06	0.57‐1.96
Dysmenorrhea or premenstrual symptoms affected their work	No night shifts	Reference		Reference	
	Two rotating shifts	1.06	0.96‐1.16	1.10	0.97‐1.23
	Three rotating shifts	1.05	0.92‐1.19	1.09	0.94‐1.27
Often affected	No night shifts	Reference		Reference	
	Two rotating shifts	1.24	0.88‐1.74	1.31	0.90‐1.90
	Three rotating shifts	1.18	0.75‐1.85	1.26	0.77‐2.07

Note

Model 1: adjusted for age. Model 2: adjusted for age, body mass index, hospital size, and the department in which the women worked.

Abbreviations: CI, confidence interval; PR, prevalence ratio.

**TABLE 3:** Association of frequency of night shifts per month and menstrual‐related variables in women working two rotating shifts

	Frequency of night shifts per month	Model 1		Model 2	
		PR	95% CI	PR	95% CI
Irregular menstrual cycles	None	Reference		Reference	
	1‐3 nights	1.27	0.92‐1.74	1.28	0.91‐1.80
	4‐5 nights	1.40	1.12‐1.75	1.41	1.08‐1.83
	≥6 nights	1.72	1.34‐2.20	1.78	1.34‐2.35
Amenorrhea ≥3 mo	None	Reference		Reference	
	1‐3 nights	0.81	0.26‐2.50	0.75	0.26‐2.22
	4‐5 nights	1.37	0.70‐2.66	1.20	0.62‐2.31
	≥6 nights	2.58	1.32‐5.03	2.39	1.27‐4.50
Abnormal menstrual period	None	Reference		Reference	
	1‐3 nights	0.87	0.41‐1.86	0.73	0.33‐1.61
	4‐5 nights	0.87	0.50‐1.49	0.69	0.38‐1.23
	≥6 nights	0.97	0.51‐1.85	0.81	0.41‐1.60
Dysmenorrhea or premenstrual symptoms affected their work	None	Reference		Reference	
	1‐3 nights	1.01	0.87‐1.18	1.04	0.88‐1.23
	4‐5 nights	1.07	0.97‐1.19	1.11	0.98‐1.26
	≥6 nights	1.04	0.91‐1.19	1.09	0.94‐1.27
Often affected	None	Reference		Reference	
	1‐3 nights	1.09	0.63‐1.88	1.15	0.66‐2.00
	4‐5 nights	1.27	0.88‐1.84	1.34	0.90‐2.00
	≥6 nights	1.26	0.80‐2.00	1.33	0.81‐2.16

Note

Model 1: adjusted for age. Model 2: adjusted for age, body mass index, hospital size, and the department in which the women worked.

Abbreviations: CI, confidence interval; PR, prevalence ratio.

**TABLE 4:** Association of frequency of night shifts per month and menstrual‐related variables in women with three rotating shifts

	Frequency of night shifts per month	Model 1		Model 2	
		PR	95% CI	PR	95% CI
Irregular cycles	None	Reference		Reference	
	≤5 nights	1.84	1.29‐2.61	1.93	1.29‐2.89
	6‐7 nights	1.47	0.95‐2.29	1.51	0.92‐2.48
	≥8 nights	1.14	0.79‐1.64	1.24	0.81‐1.89
Amenorrhea ≥3 mo	None	Reference		Reference	
	≤5 nights	2.50	0.94‐6.65	3.84	1.21‐12.22
	6‐7 nights	1.70	0.52‐5.59	2.07	0.58‐7.47
	≥8 nights	0.45	0.10‐1.99	0.75	0.15‐3.66
Abnormal menstrual period	None	Reference		Reference	
	≤5 nights	0.76	0.24‐2.43	0.74	0.22‐2.50
	6‐7 nights	1.72	0.70‐4.19	1.55	0.53‐4.51
	≥8 nights	1.41	0.69‐2.89	1.32	0.60‐2.89
Dysmenorrhea or premenstrual symptoms affected their work	None	Reference		Reference	
	≤5 nights	1.09	0.90‐1.33	1.19	0.96‐1.48
	6‐7 nights	0.91	0.69‐1.20	0.98	0.73‐1.33
	≥8 nights	1.12	0.96‐1.31	1.23	1.02‐1.49
Often affected	None	Reference		Reference	
	≤5 nights	0.77	0.31‐1.93	1.00	0.36‐2.74
	6‐7 nights	0.98	0.43‐2.24	1.20	0.47‐3.06
	≥8 nights	1.51	0.90‐2.53	1.93	1.03‐3.61

Note

Model 1: adjusted for age. Model 2: adjusted for age, body mass index, and hospital size, and the department in which women worked.

Abbreviations: CI, confidence interval; PR, prevalence ratio.

A total of 115 (8.4%) women who took oral contraceptives were excluded from the main analysis. Their reasons for taking oral contraceptives included contraception (37/130, 28.5%), dysmenorrhea (61/130, 46.9%), irregular menstrual cycles (57/130, 43.9%), treatment for endometriosis (21/130, 16.2%), and others (14/130, 10.8%); there were overlapping reasons.

## 4 DISCUSSION

This cross‐sectional study demonstrated a relationship between night shifts and irregular menstrual cycles among Japanese nurses. A dose‐response relationship was observed between the frequency of night shifts per month and irregular menstrual cycles among women working under two rotating shifts. This result is consistent with a previous study that included a large sample size.^8^ Among women working two rotating shifts, night shift of ≥6 night shifts per month was associated with both irregular menstrual cycles and secondary amenorrhea. This implies that high frequency of night shifts might increase the risk of adverse health outcomes.

Although short menstruation (1‐2 days) and prolonged menstrual period (≥8 days) are not normal,^20^ the relationship between shift work and menstrual period has not been sufficiently evaluated compared with menstrual cycle length and regularity. This study showed that the menstrual period was not affected by the frequency of night shifts; however, low prevalence of abnormal menstrual period and the possibility that women with abnormal menstrual periods were taking oral contraceptives as treatment might make it difficult to evaluate the effect of shift work on menstrual period.

In this study, more than 60% of women responded that dysmenorrhea or premenstrual symptoms affected their work, and approximately 10% of women responded that those symptoms often affected their work. This was consistent with previous research, which showed that concentration at school and participation in social events were affected in about 60% of adolescent girls.^21^ It might be difficult to infer the association between the frequency of night shifts and effect of dysmenorrhea or premenstrual symptoms on their work from this study because of the inconsistency of results in women working two and three rotating shifts. The previous study reported that 76.1% of women believed that dysmenorrhea is a part of the natural course of menstrual cycle and only 14.8% of the women believed that treatment was necessary.^21^ Therefore, further studies focusing on the treatment situation and women's attitude toward treatment are required.

A meta‐analysis, including 123 403 women, reported that the prevalence of irregular menstrual cycle in female shift workers was 16.05%.^22^ In contrast, the PR of irregular menstrual cycle in women working rotating shifts was comparatively higher (approximately 30%‐40%) in this study. Although cohort studies from China and Taiwan reported that the PR of irregular menstrual cycles were 24.3%‐45.8%, these studies included women working three rotating shifts.^23^, ^24^ Our result is consistent with the results in other Asian countries; however, in the present study, most women worked under two rotating night shifts. A hospital nursing survey in 2019 reported that 66.5% of hospitals adapted two rotating shifts, and most adapted the 8‐16 hours shift in Japan.^16^ Many countries adapted the 12‐12 hours shift as the two rotating shifts; hence, the health risk of this irregular two rotating shift has not been sufficiently evaluated. In addition to ethnicities, heavy workloads and high stress levels increase the risk of irregular menstrual cycles.^9^, ^25^ Although further analyses of workloads and stress levels will be necessary, the results of this study imply that irregular two rotating shifts can increase the risk of irregular menstrual cycle similarly to three rotating shifts.

In contrast, a dose‐response relationship was not observed between the frequency of night shifts per month and irregular menstrual cycles in women working three rotating shifts. Three rotating shifts comprise a day shift, evening shift, and night shift. The number of night shifts worked was asked in the questionnaire; however, the frequency of night shifts tended to be higher in women working three rotating shifts than in those with two rotating shifts. This result suggests that the frequency of night shifts in women working three rotating shifts included both evening and night shifts in this study. A previous study from China reported that the frequency of evening shift did not increase the risk of irregular menstrual cycle.^24^ Hence, contamination of the evening shift might be a reason why a dose‐response relationship was not observed in women working three rotating shifts.

There were several limitations in this study. First, the risk of irregular menstrual cycle might have been underestimated because we excluded women who were taking oral contraceptives; 43.9% of the women took oral contraceptives because of irregular menstrual cycle. Second, the study design is a cross‐sectional design; therefore, we cannot exclude the effect of recall bias. Third, selection bias could have been a problem due to the low response rate. In contrast, the questionnaire used in this study included questions on both irregular menstrual cycles and nursing education. This can reduce the risk of overestimating irregular menstrual cycle because women worried about irregular menstrual cycle might experience irregular menstrual cycles more frequently compared with those who did not worry. In addition, participant's educational and financial backgrounds were relatively homogenous, and they had a high literacy regarding web information because only nurses were enrolled and questionnaire surveys are often conducted on “*Nurse senka plus*”. Fourth, no information was collected about past history of gynecological disease, pregnancy, and workload. Therefore, the effects of these factors on menstrual disorders could not be determined in this study. Finally, irregular menstrual cycle was analyzed by a self‐reported questionnaire and misclassification cannot be ruled out. However, a previous validation study in non‐medical staff reported that ≥ 50% of the women in their study revealed that their usual cycle length was within 2 days of their mean menstrual cycle length.^26^ Although a validation study of the self‐reported questionnaire on menstrual cycle in healthcare workers has not been conducted, we assumed that healthcare workers were able to report their menstrual cycles more accurately than non‐healthcare workers. In addition, the similarity between the results of this studies compared with those of previous studies validates the data collection in this study. Menstrual diaries can provide more accurate information regarding menstrual cycle and symptoms associated with menstruation. Therefore, a future prospective study using menstrual diaries will contribute toward reducing the risk of misclassification.

## 5 CONCLUSION

In the present study, the prevalence of irregular menstrual cycles among Japanese female nurses working under rotating shifts was estimated. The frequency of night shifts had a dose‐response relationship with irregular menstrual cycles and a total of at least six night shifts per month increased the risk of secondary amenorrhea in women working two rotating shifts. Night work is inevitable in the nursing profession; therefore, interventions for reducing the risk of irregular menstrual cycles are necessary for protecting the health of these working women.

## DISCLOSURE

*Approval of the research protocol*: The institutional review board of Shimane University approved this study (20181018‐2). *Informed consent*: Completion of the web‐based questionnaires implied informed consent. *Registry and Registration No*: N/A. *Animal studies*: N/A. *Conflict of interest*: The authors have no conflict of interest with respect to this study.

## AUTHOR CONTRIBUTIONS

Michinori Mayama, Hidemichi Watari, and Takashi Watari conceived the ideas; Michinori Mayama, Sho Nishiguchi, and Takuhiro Moromizato collected the data; Michinori Mayama and Takashi Umazume analyzed the data; Michinori Mayama led the writing; all the authors revised the manuscript and approved the final version of the manuscript.

## Supporting information

Table S1‐S2

## References

[1] Baker FC , Driver HS . Circadian rhythms, sleep, and the menstrual cycle. Sleep Med. 2007;8:613‐622. 17383933 10.1016/j.sleep.2006.09.011

[2] Reindollar RH , Novak M , Tho SP , McDonough PG . Adult‐onset amenorrhea: a study of 262 patients. Am J Obstet Gynecol. 1986;155:531‐543. 3529965 10.1016/0002-9378(86)90274-7

[3] Marcus MD , Loucks TL , Berga SL . Psychological correlates of functional hypothalamic amenorrhea. Fertil Steril. 2001;76:310‐316. 11476778 10.1016/s0015-0282(01)01921-5

[4] Solomon CG , Hu FB , Dunaif A , et al. Menstrual cycle irregularity and risk for future cardiovascular disease. J Clin Endocrinol Metab. 2002;87:2013‐2017. 11994334 10.1210/jcem.87.5.8471

[5] Meczekalski B , Podfigurna‐Stopa A , Genazzani AR . Hypoestrogenism in young women and its influence on bone mass density. Gynecol Endocrinol. 2010;26:652‐657. 20504098 10.3109/09513590.2010.486452

[6] Harlow SD , Ephross SA . Epidemiology of menstruation and its relevance to women's health. Epidemiol Rev. 1995;17:265‐286. 8654511 10.1093/oxfordjournals.epirev.a036193

[7] Harlow SD , Campbell OM . Epidemiology of menstrual disorders in developing countries: a systematic review. BJOG. 2004;111:6‐16. 14687045 10.1111/j.1471-0528.2004.00012.x

[8] Lawson CC , Whelan EA , Lividoti Hibert EN , Spiegelman D , Schernhammer ES , Rich‐Edwards JW . Rotating shift work and menstrual cycle characteristics. Epidemiology. 2011;22:305‐312. 21364464 10.1097/EDE.0b013e3182130016 PMC5303197

[9] Lawson CC , Johnson CY , Chavarro JE , et al. Work schedule and physically demanding work in relation to menstrual function: the Nurses' Health Study 3. Scand J Work Environ Health. 2015;41:194‐203. 25634477 10.5271/sjweh.3482

[10] Kim JJ , Choi YM . Phenotype and genotype of polycystic ovary syndrome in Asia: Ethnic differences. J Obstet Gynaecol Res. 2019;45:2330‐2337. 31588677 10.1111/jog.14132

[11] Gu BH , Park JM , Baek KH . Genetic variations of follicle stimulating hormone receptor are associated with polycystic ovary syndrome. Int J Mol Med. 2010;26:107‐112. 20514429 10.3892/ijmm_00000441

[12] Kim JJ , Choi YM , Hong MA , et al. FSH receptor gene p. Thr307Ala and p. Asn680Ser polymorphisms are associated with the risk of polycystic ovary syndrome. J Assist Reprod Genet. 2017;34:1087‐1093. 28547204 10.1007/s10815-017-0953-z PMC5533683

[13] Fernandez H , Barea A , Prevalence C‐L . Intensity, impact on quality of life and insights of dysmenorrhea among French women: a cross‐sectional web survey. J Gynecol Obstet Hum Reprod. 2020;101889 (Online ahead of print). 32781307 10.1016/j.jogoh.2020.101889

[14] Chiu MH , Hsieh HF , Yang YH , Chen HM , Hsu SC , Wang HH . Influencing factors of dysmenorrhoea among hospital nurses: a questionnaire survey in Taiwan. BMJ Open. 2017;7:e017615. 10.1136/bmjopen-2017-017615 PMC5778302 29259057

[15] Albert‐Sabater JA , Martínez JM , Baste V , Moen BE , Ronda‐Perez E . Comparison of menstrual disorders in hospital nursing staff according to shift work pattern. J Clin Nurs. 2016;25:3291‐3299. 27530371 10.1111/jocn.13371

[16] Japanese Nursing Association . “2019 Byouinkango zittaichousa. nihonkangokyoukai tyousakennkyuhoukoku” [2019 Hospital Nursing Survey. Japanese Nursing Association Research Reports]. https://www.nurse.or.jp/home/publication/pdf/research/95.pdf. Accessed April 21, 2020 (in Japanese)

[17] Tamada T , Iwasaki H . Age at natural menopause in Japanese women. Acta Obstet Gynecol Jpn. 1995;47:947‐952. (in Japanese) 7594906

[18] Samar R , Khoudary EL , Greendale G , et al. The menopause transition and women's health at midlife: a progress report from the Study of Women's Health Across the Nation (SWAN). Menopause. 2019;26:1213‐1227. 31568098 10.1097/GME.0000000000001424 PMC6784846

[19] Japan Society of Obstetrics and Gynecology . “sankafuzinka yougosyu yougokaisetsusyu kaitei dai 4 han” [Terminology of Obstetrics and Gynecology revised 4th edition]. *Kanehara shuppan*; 2018. (in Japanese).

[20] Maybin JA , Critchley HO . Menstrual Physiology: implications for endometrial pathology and beyond. Hum Reprod Update. 2015;21:748‐761. 26253932 10.1093/humupd/dmv038 PMC4594618

[21] Wong LP . Attitudes towards dysmenorrhoea, impact and treatment seeking among adolescent girls: a rural school‐based survey. Aust J Rural Health. 2011;19:218‐223. 21771164 10.1111/j.1440-1584.2011.01213.x

[22] Stocker LJ , Macklon NS , Cheong YC , Bewley SJ . Influence of shift work on early reproductive outcomes: a systematic review and meta‐analysis. Obstet Gynecol. 2014;124:99‐110. 24901274 10.1097/AOG.0000000000000321

[23] Chung FF , Yao CC , Wan GH . The associations between menstrual function and lifestyle/working conditions among nurses in Taiwan. J Occup Health. 2005;47:149‐156. 15824480 10.1539/joh.47.149

[24] Wang Y , Gu F , Deng M , et al. Rotating shift work and menstrual characteristics in a cohort of Chinese nurses. BMC Women's Health. 2016;16:24. 27145834 10.1186/s12905-016-0301-y PMC4857333

[25] Hatch MC , Figa‐Talamanca I , Salerno S . Work stress and menstrual patterns among American and Italian nurses. Scand J Work Environ Health. 1999;25:144‐150. 10360470 10.5271/sjweh.417

[26] Small CM , Manatunga AK , Marcus M . Validity of self‐reported menstrual cycle length. Ann Epidemiol. 2007;17:163‐170. 16882471 10.1016/j.annepidem.2006.05.005

