# Supplementary material for: Frequency of night shift and menstrual cycle characteristics in Japanese nurses working under two or three rotating shifts
Source: J Occup Health. 2020 Nov 19;62(1):e12180. doi: 10.1002/1348-9585.12180 (PMC7676323; doi:10.1002/1348-9585.12180)
Supplement: Supplementary file 1 — Table S1‐S2 [file JOH2-62-e12180-s001.docx]

Table S1. Characteristics and menstrual-related variables stratified by the frequency of night shifts per month over the past year in women working two rotating shifts.

|  | Frequency of night shifts per month in two rotating shifts women | | | |
| --- | --- | --- | --- | --- |
|  | 1–3 nights  (n = 123) | 4–5 nights  (n = 392) | ≥6 nights  (n = 164) | p-value |
| **Age (y.o)** |  |  |  |  |
| <30 | 51 (41.5) | 167 (42.6) | 56 (34.2) | 0.090 |
| ≥30 and <40 | 32 (26.0) | 132 (33.7) | 62 (37.8) |  |
| ≥40 and <45 | 40 (32.5) | 93 (23.7) | 46 (28.1) |  |
| **BMI (kg/m^2^)** |  |  |  |  |
| ≥18.5 and <25 | 94 (76.4) | 281 (71.7) | 109 (66.5) | 0.127 |
| <18.5 | 9 (7.3) | 55 (14.0) | 33 (20.1) |  |
| ≥25 and <30 | 14 (11.4) | 43 (11.0) | 16 (9.8) |  |
| >30 | 6 (4.5) | 13 (3.3) | 6 (3.7) |  |
| **Hospital size** |  |  |  |  |
| >300 beds | 75 (61.0) | 216 (55.1) | 90 (54.9) | 0.417 |
| 100-299 beds | 37 (30.1) | 135 (34.4) | 54 (32.9) |  |
| <100 beds | 4 (3.3) | 27 (6.9) | 9 (5.5) |  |
| Clinic, nursing home | 7 (5.7) | 14 (3.6) | 11 (6.7) |  |
| **The department women worked in** |  |  |  |  |
| Out-patient clinic | 18 (14.6) | 29 (7.4) | 16 (9.8) | <0.001 |
| Wards | 87 (70.7) | 331 (84.4) | 112 (68.3) |  |
| ICU, OR, ER | 18 (14.6) | 32 (8.2) | 36 (22.0) |  |
| **Irregular menstrual cycles ^*^** | 40 (32.5) | 142 (36.2) | 72 (43.9) | 0.108 |
| cycle length ≤21 days or ≥39 days at least a few times | 36 (29.3) | 120 (30.6) | 56 (34.2) | 0.107 |
| amenorrhea ≥3 months | 4 (3.3) | 22 (5.6) | 16 (9.8) |  |
| **Abnormal menstrual period^†^** | 8 (6.5) | 25 (6.4) | 12 (7.3) | 0.919 |
| 1–2 days | 3 (2.4) | 6 (1.5) | 6 (3.7) | 0.587 |
| ≥8 days | 5 (4.1) | 19 (4.9) | 6 (3.7) |  |
| **Dysmenorrhea or premenstrual symptoms affected their work^‡^** | 80 (65.0) | 273 (69.6) | 110 (67.1) | 0.595 |
| sometimes | 66 (53.7) | 225 (57.4) | 88 (53.7) | 0.849 |
| often | 14 (11.4) | 48 (12.2) | 22 (13.4) |  |

Data are shown in n (%).

BMI: body mass index , ICU: intensive care unit, OR: operating room, ER: emergency room

Statistical significance was calculated using chi-square test.

*: normal menstrual cycle length is >22 days and <39 days.

†: normal menstrual period is 3–7 days.

‡: dysmenorrhea and premenstrual symptoms do not affect their work in a normal person.

Table S2. Characteristics and menstrual-related variables stratified by the frequency of night shifts per month over the past year in women working three rotating shifts.

|  | Frequency of night shifts per month in three rotating shifts women | | | |
| --- | --- | --- | --- | --- |
|  | ≤5 nights  (n = 56) | 6–7 nights  (n = 40) | ≥8 nights  (n = 99) | p-value |
| **Age (y.o)** |  |  |  |  |
| <30 | 28 (50.0) | 16 (40.0) | 41 (41.4) | 0.098 |
| ≥30 and <40 | 21 (37.5) | 13 (32.5) | 26 (26.3) |  |
| ≥40 and <45 | 7 (12.5) | 11 (27.5) | 32 (32.3) |  |
| **BMI (kg/m^2^)** |  |  |  |  |
| ≥18.5 and <25 | 43 (76.8) | 30 (75.0) | 77 (77.8) | 0.365 |
| <18.5 | 10 (17.9) | 4 (10.0) | 12 (12.1) |  |
| ≥25 and <30 | 2 (3.6) | 2 (5.0) | 7 (7.1) |  |
| >30 | 1 (1.8) | 4 (10.0) | 3 (3.0) |  |
| **Hospital size** |  |  |  |  |
| >300 beds | 34 (60.7) | 23 (57.5) | 62 (62.6) | 0.609 |
| 100-299 beds | 18 (32.1) | 16 (40.0) | 30 (30.3) |  |
| <100 beds | 3 (5.4) | 1 (2.5) | 7 (7.1) |  |
| Clinic, nursing home | 1 (1.8) | 0 | 0 |  |
| **The department women worked in** |  |  |  |  |
| Out-patient clinic | 3 (5.4) | 2 (5.0) | 3 (3.0) | 0.796 |
| Wards | 46 (82.1) | 33 (82.5) | 78 (78.8) |  |
| ICU, OR, ER | 7 (12.5) | 5 (12.5) | 18 (18.2) |  |
| **Irregular menstrual cycles ^*^** | 26 (46.4) | 15 (37.5) | 29 (29.3) | 0.099 |
| cycle length ≤21 days or ≥39 days at least a few times | 20 (35.7) | 12 (30.0) | 27 (27.3) | 0.107 |
| amenorrhea ≥3 months | 6 (10.7) | 3 (7.5) | 2 (2.0) |  |
| **Abnormal menstrual period^†^** | 3 (5.4) | 5 (12.5) | 10 (10.1) | 0.449 |
| 1–2 days | 1 (1.8) | 2 (5.0) | 1 (1.0) | 0.418 |
| ≥8 days | 2 (3.6) | 3 (7.5) | 9 (9.1) |  |
| **Dysmenorrhea or premenstrual symptoms affected their work^‡^** | 39 (69.6) | 23 (57.5) | 70 (70.7) | 0.300 |
| sometimes | 35 (62.5) | 18 (45.0) | 55 (55.6) | 0.310 |
| often | 4 (7.1) | 5 (12.5) | 15 (15.2) |  |

Data are shown in n (%).

BMI: body mass index, ICU: intensive care unit, OR: operating room, ER: emergency room

Statistical significance was calculated using chi-square test.

*: normal menstrual cycle length is >22 days and <39 days.

†: normal menstrual period is 3–7 days.

‡: dysmenorrhea and premenstrual symptoms do not affect their work in a normal person.
